# Electrometer offset current due to scattered radiation

**DOI:** 10.1002/acm2.12458

**Published:** 2018-10-09

**Authors:** Sonja Wegener, Otto A. Sauer

**Affiliations:** ^1^ Department of Radiation Oncology University of Wuerzburg Wuerzburg Germany

**Keywords:** electrometer, micro‐ionization chambers, polarity, relative dosimetry, scatter radiation

## Abstract

Relative dose measurements with small ionization chambers in combination with an electrometer placed in the treatment room (“internal electrometer”) show a large dependence on the polarity used. While this was observed previously for percent depth dose curves (PDDs), the effect has not been understood or preventable. To investigate the polarity dependence of internal electrometers used in conjunction with a small‐volume ionization chamber, we placed an internal electrometer at a distance of 1 m from the isocenter and exposed it to different amounts of scattered radiation by varying the field size. We identified irradiation of the electrometer to cause a current of approximately −1 pA, regardless of the sign of the biasing voltage. For low‐sensitivity detectors, such a current noticeably distorts relative dose measurements. To demonstrate how the current systematically changes PDDs, we collected measurements with nine ionization chambers of different volumes. As the chamber volume decreased, signal ratios at 20 and 10 cm depth (M20/M10) became smaller for positive bias voltage and larger for negative bias voltage. At the size of the iba CC04 (40 mm³) the difference of M20/M10 was around 1% and for the smallest studied chamber, the iba CC003 chamber (3 mm³), around 7% for a 10 × 10 cm² field. When the electrometer was moved further from the source or shielded, the additional current decreased. Consequently, PDDs at both polarities were brought into alignment at depth even for the 3 mm³ ionization chamber. The apparent polarity effect on PDDs and lateral beam profiles was reduced considerably by shielding the electrometer. Due to normalization the effect on output values was low. When measurements with a low‐sensitivity probe are carried out in conjunction with an internal electrometer, we recommend careful monitoring of the particular setup by testing both polarities, and if deemed necessary, we suggest shielding the electrometer.

## 1 INTRODUCTION

Many different detectors are available for small field dosimetry, for example, for output factor measurements or beam profile and percent depth dose curves (PDD) acquisitions. Ionization chambers are frequently used for such measurements in radiation therapy. Since larger active detector volumes can lead to volume averaging effects, ionization chambers have recently been produced with an active volume as small as 3 mm³.^1^

The sensitivity of such a detector drops with its volume, thereby resulting in a worse signal‐to‐noise ratio for chambers with smaller active volume. In addition, small ionization chambers are susceptible to effects that have not been observed for larger chambers, including effects on measured PDDs. Discrepancies in measurements of PDDs with small‐volume ionization chambers have been observed by Sarkar et al.^2^ They noticed PDD discrepancies at different polarities when measuring with an electrometer placed in the treatment room, which were not present with an electrometer placed outside of the treatment room. Although they ruled out a number of influence parameters, it remained unclear where exactly the interference was originating.

Polarity effects manifest themselves as different readings between a positive and a negative bias voltage. For micro‐ionization chambers, this effect is voltage dependent and increases as their volumes decrease due to higher relative changes in the collecting volume.^3^ TRS 483 demands a polarity effect of less than 0.4% of the chamber reading as a specification for reference class ionization chambers for absolute dosimetry.^4^ The same maximum polarity correction of 0.4% from unity at any energy is recommended in the addendum to the TG‐51 protocol.^5^ Smaller chambers often do not meet this criterion; for example, according to Hyun et al. the mean polarity correction factor averaged over several beam qualities at 5 cm depth in a 10 × 10 cm² field for a CC01 (10 mm³) micro‐chamber was 1.011.^6^ While certain chambers have shown depth‐dependent polarity effects,^7^, ^8^, ^9^, ^10^ the anomalous PDD behavior observed by Sarkar et al. for small‐volume chambers vanished when using an external electrometer, suggesting it was likely not caused by the chamber or the polarity effect.

We encountered similar problems to Sarkar et al. when scanning PDDs at either a positive or negative bias with the electrometer placed inside the treatment room. This type of electrometer setup will be referred to as an “internal electrometer”; while an electrometer placed outside of the treatment room will be referred to as an “external electrometer”. We investigated the cause of this effect and present ways to mitigate and correct it to increase the accuracy of PDDs, profiles, and output factors obtained using small‐volume chambers.

## 2 MATERIALS AND METHODS

### 2.A Radiation effects on an internal electrometer

**Figure 1:**
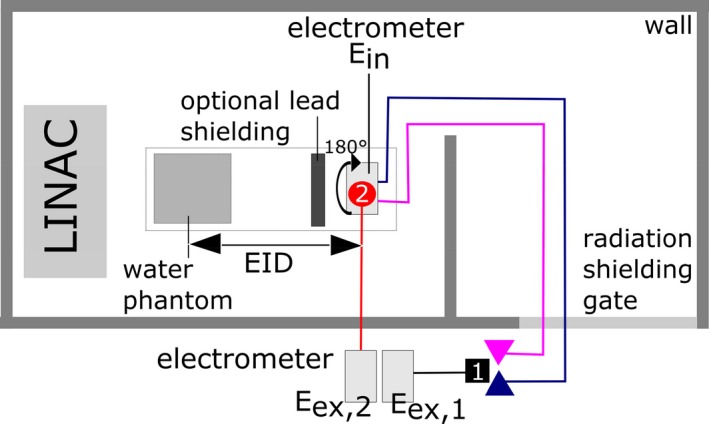
Measurement setup. Two ionization chambers were placed outside of the treatment room (Point 1 external) and connected to an internal electrometer placed at a distance *EID* from the isocenter (Field Channel, Reference Channel). A third detector was placed next to the first two detectors (Point 1 external) and connected to an external electrometer outside the treatment room. A fourth detector was placed on top of the internal electrometer (Point 2 internal) and connected to an external electrometer. A water‐filled phantom was placed at the isocenter. In some experiments, lead bricks were placed between the electrometer and the phantom or the internal electrometer was rotated by 180°.

To evaluate the origin of the observed deviations, two ionization chambers were connected to a Tandem internal electrometer (PTW, Germany). A Semiflex 31013 chamber (PTW, Germany, 0.3 cm³) was connected to the field channel while a Famer type 30013 (PTW, Germany, 0.6 cm³) was connected to the reference channel. Using two 8 m cables, the chambers were positioned just outside of the treatment room with the doors closed. The electrometer was positioned on the treatment couch at a certain electrometer‐to‐isocenter distance *EID* (Fig. 1). In addition, a second Semiflex 31013 was placed next to the other chambers outside the treatment room door and connected to a Unidos external electrometer (PTW, Germany). A third Semiflex 31013 was inserted into a plastic cube, put on top of the internal electrometer and connected to a Unidos E electrometer (PTW, Germany) outside the treatment room. To generate realistic scatter conditions, a MP3‐XS phantom (PTW, Germany) was filled with water and placed as if measurements at a SSD of 100 cm were carried out. The MP3‐XS has a volume of 34 × 34 × 42 cm³.

A 6 MV beam from a Primus linac (Siemens, Germany) was used for the measurements. The linac operates at a repetition rate of 250 MU/min (±1%), and is calibrated to deliver 1 cGy/MU at the isocenter at a depth of 5 cm in water. All four chamber signals were zeroed while the beam was off. For the following measurements, the current was integrated over 30 s for all chambers nearly simultaneously. The integrated value was read directly from the Unidos electrometers’ display and obtained from the water tank software (Mephysto mc² TanSoft, PTW, Germany, version 1.4) for the internal electrometer. Measurements were repeated 3 to 5 times. A bias voltage of 400 V was used for all chambers. All measurements were repeated with a negative bias voltage applied to the internal electrometer. After the bias voltage was changed, the current was stabilized before the measurements were continued.

Measurements at a constant electrometer to isocenter distance *EID* = 100 cm were carried out for different nominal field sizes from 1 × 1 to 20 × 20 cm², as well as for beam off, using both polarities. Measurements were then carried out for a 10 × 10 cm² field at the *EID* of 100 and 150 cm, and additionally for the latter with 10 cm of lead as a shielding between the electrometer and the water phantom**.** Note that the cables were not shielded explicitly. At *EID* = 150 cm, the phantom is positioned at the head end of the table and the electrometer at the feet end to achieve the largest distance that still fits the electrometer on the treatment couch. Finally, at an *EID* = 100 cm and a field size of 10 × 10 cm², the effects of exchanging the chambers between the reference and the field channel as well as rotating the electrometer by 180 degrees were tested. To rule out the malfunction of the used individual device, measurements for this configuration were also repeated with a second Tandem electrometer at both polarities.

### 2.B Depth dose curves

Depth dose curves for a 10 × 10 cm² field were measured in an MP3‐M (PTW, Germany) water phantom at a source to surface distance (SSD) of 100 cm. While all other measurements could be performed with the small tank, a larger size tank was preferable for the longer Farmer type detectors. Therefore, the large water phantom MP3‐M having dimensions of 55 × 55 × 64 cm³ was used for the comparison of different detectors. Measurements were performed using the internal Tandem electrometer and the corresponding acquisition software (Mephysto mc² tbaScan, PTW, Germany, version 3.2). To mimic the typical convenient setup, the electrometer was located in the treatment room on the couch at an *EID* of 150 cm.

Ionization chambers of different construction types and volumes were used (Table 1): Plane‐parallel chambers of the Roos PPC40 and Markus PPC05 type, Farmer type chambers FC65‐G and the shorter version FC23C, thimble ionization chamber CC13, and micro‐chambers iba CC04, CC01 and CC003 (all iba dosimetry, Germany) as well as a PTW PinPoint 31006. The chamber position in the field center was verified by acquiring lateral profiles. Applied voltages were ±300 V for iba chambers and ±400 V for the PTW chamber according to the manufacturer recommendations. Before each measurement, including every change in the polarity, chambers were pre‐irradiated to approximately 5 to 10 Gy depending on the chamber size. To account for output variations, a transmission chamber T‐REF (PTW, Germany) below the collimator was used as a reference detector.

**Table 1:** Data for all detectors used to obtain depth dose curves according to manufacturer information

Detector	Volume (cm³)	Detector type	Radius of active volume (mm)	Length of active volume (mm)
FC 65‐G	0.65	Farmer	3.1	23
FC23	0.23	Farmer	3.1	9
CC13	0.13	Scanning	3.0	5.8
CC04	0.04	Micro	2.0	3.6
PinPoint 31006	0.015	Micro	1	5
CC01	0.01	Micro	1.0	3.6
CC003	0.003	Micro	1.0	2.0
PPC05	0.05	Markus	4.95	0.6
PPC40	0.40	Roos	8	2

The M20/M10 ratio of the relative dose at 20 and 10 cm depth were calculated for all detectors and both polarities. The effective point of measurement was taken into account by shifting the cylindrical chamber positions by r/2 from the chamber axes and choosing the reference point of the plane‐parallel chambers as 0.4 mm behind the entry window.

To quantitate the effect of the offset current on relative dose measurements, the depth dose, lateral profile, and relative output factor measurements were performed at different internal electrometer positions. All of the following scanning measurements were carried out using the small MP3‐XS phantom and the same electrometer and software described previously in this section. For the smallest chamber, the CC003, depth dose curves were measured at *EID* = 100 cm and *EID* = 150 cm for both polarities. In addition, at EID = 150 cm the Tandem electrometer was shielded. For comparison, a conventional scanning chamber, the CC13, was used in the setup with the shielded electrometer.

### 2.C Profile measurements

Lateral beam profiles at SSD 90 cm at a depth of 10 cm were taken at a nominal 8 × 8 cm² field at both polarities with a CC003 chamber oriented perpendicular to the beam axis. Scans were collected in plane with the detector oriented perpendicular to the scan direction to avoid a scanning direction of the detector moving “tip first” resulting in a variable irradiated detector length. For comparison, profile measurements were repeated with a microDiamond 60019 detector with the electrometer positioned at *EID* = 150 cm and behind the shielding.

### 2.D Output factor measurements

Output factor measurements were taken for both polarities at an SSD 90 at 20 cm depth with the CC003 chamber for nominal square field sizes of 10, 6, 4, 2, and 1 cm, 100 MU, and three readings per field size using the TanSoft software. Directly afterwards, a Semiflex 31013 ionization chamber was placed outside the treatment room and connected to the Tandem electrometer field channel inside to quantitate the effect of scattered radiation on the electrometer in exactly the same setup for all field sizes used. Measurements were repeated three times with 100 MU. The measured charges obtained were then corrected for the electrometer offset determined individually for each field size. As the charge induced by electrometer irradiation was always negative, its absolute value was added to correct the signed electrometer readings obtained with TanSoft. Output factor measurements were also carried out at SSD 90 and 10 cm depth with the CC003 chamber either connected to the internal electrometer at *EID* = 150 cm unshielded or the Unidos external electrometer for both polarities. Fields between 10 × 10 and 0.6 × 0.6 cm² nominal size were measured three times each with 100 MU. The same measurements were repeated with a microDiamond 60019 detector connected to the external electrometer.

## 3 RESULTS

**Figure 2:**
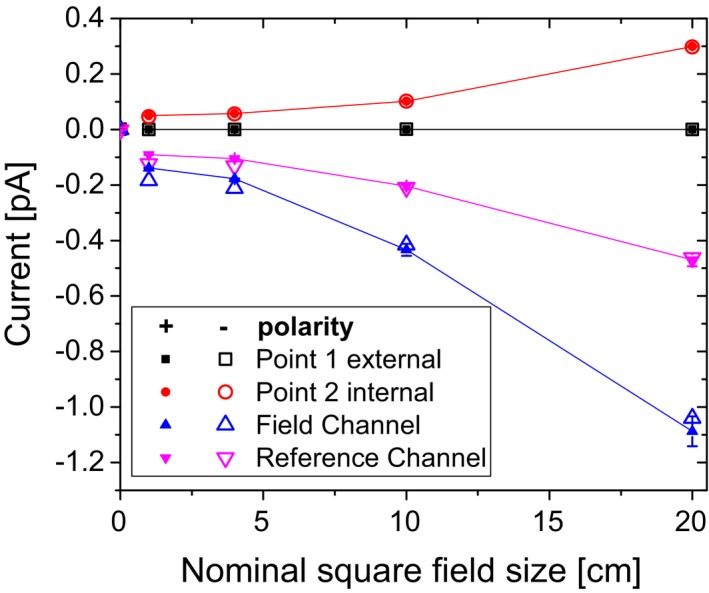
Electrometer current for all four detectors (positions see Fig. 1) for EID = 100 cm as a function of the nominal field size. Detectors positioned outside the treatment room at Point 1 (triangles) are connected to the internal electrometer with positive (solid symbols) and negative bias voltage (open symbols). The current measured at point 1 when the detector is connected to an external electrometer is displayed as boxes. The signal of a detector at Point 2 in the treatment room is displayed as circles and indicates how much the dose at the electrometer increases with field size. Error bars (only shown for positive polarity) represent one standard deviation when multiple measurements were taken with the same chamber.

Measured currents with the setup shown in Fig. 1 for different field sizes are shown in Fig. 2. The current for an ionization chamber placed outside of the treatment room connected to the Tandem electrometer E_in_ placed in the treatment room changed when the beam was on. At the same time, the current in the chamber of identical type connected to the electrometer E_ext,1_ placed outside of the treatment room did not change, indicating that the non‐zero signal was not due to the detector. The measured current was negative regardless of the polarity used.

**Figure 3:**
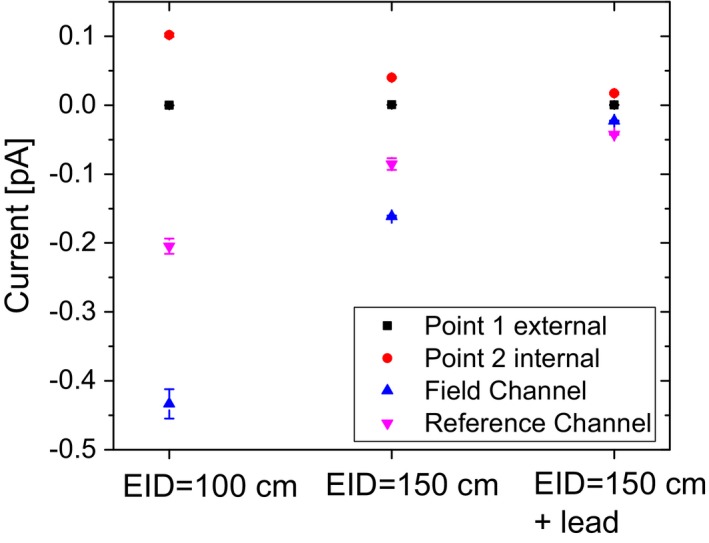
Electrometer current for all four detectors (positions see Fig. 1) as a function of the position of the internal electrometer and with additional lead shielding at positive bias voltage. Error bars represent one standard deviation when multiple measurements were taken with the same chamber.

The currents measured by the internal electrometer differed between its two channels. They were larger for larger field sizes and showed the same relative increase as the signal measured with the chamber located on top of the internal electrometer. Interchanging the detectors connected to the field and the reference channel did not noticeably change the signal obtained for each channel. The current measured with a second electrometer, identical in construction, in the same setup was comparable to the first measurement. Rotating the electrometer by 180 degrees reduced the current in both channels. Currents vs EID at a fixed field size are shown in Fig. 3. Moving the electrometer further away reduced the signal obtained from the internal electrometer. The lead shielding resulted in a substantial reduction in the current.

**Figure 4:**
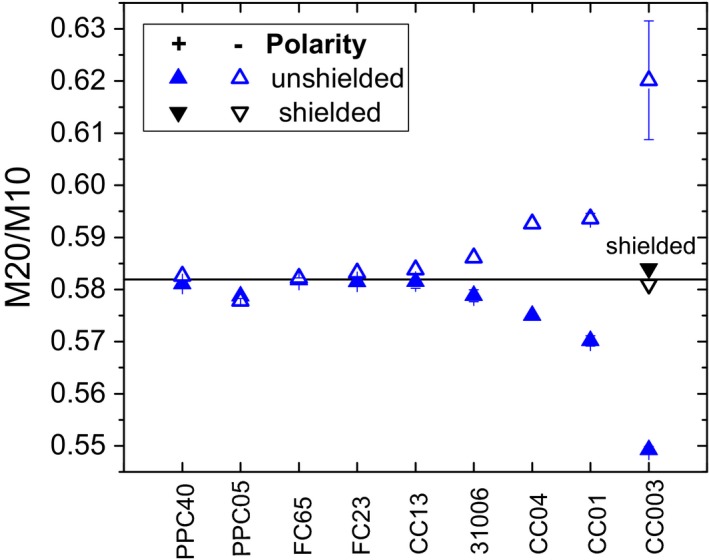
Ratio of relative signals at 20 and 10 cm depth for different detectors at positive and negative bias voltage measured with an internal electrometer without additional shielding. Chambers are sorted according to their active volume. Horizontal line indicates the mean of PPC40 and FC65 at both polarities. Error bars represent one standard deviation when multiple measurements were taken with the same chamber.

Figure 4 shows the signal ratio at two measurement depths, M20/M10, for different detectors ordered by their volume. For the larger chambers down to the size of the CC13, there was no noticeable difference between the polarities of a chamber and all chambers also yielded approximately the same M20/M10. Below the size of the CC13, it can be observed that the smaller the detector, the larger the difference between the two polarities used. The mean between positive and negative bias was closer to the value obtained with the larger chambers than each individual polarity. For the smallest CC003 chamber, the M20/M10 value was 7% lower for the positive than for the negative bias voltage. With the added shielding, the difference decreased to 0.3%.

**Figure 5:**
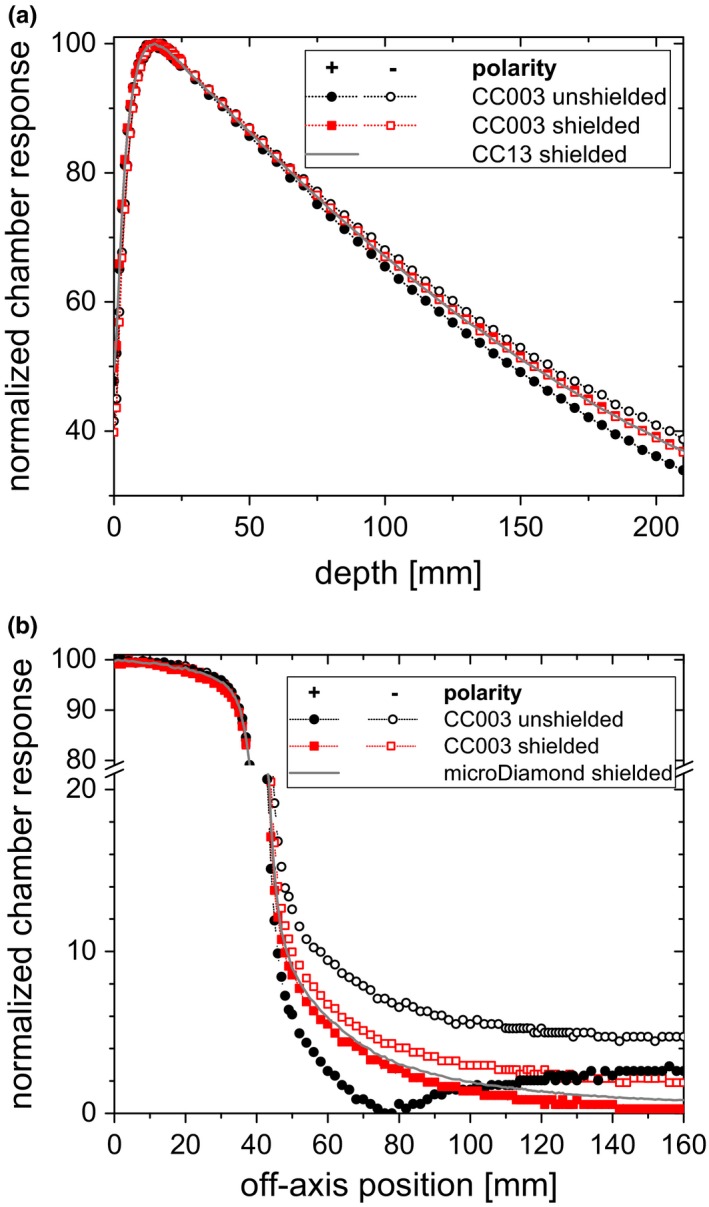
Depth dose curves (a) and lateral beam profiles (b) measured with the CC003 chamber at positive and negative bias voltage with and without additional lead shielding. Curves measured with a larger CC13 ionization chamber and a microDiamond, respectively, are shown for comparison.

Figure 5(a) shows the depth dose curves measured with the CC003 chamber for both polarities at *EID* = 150 cm. The difference between the relative chamber signals at opposing polarities at 20 cm depth was approximately 5% when measured with the unshielded electrometer. The same curves measured with the lead shielding in place did not show the deviation between the polarities at depths deeper than the depth of maximum dose. The difference at 20 cm depth was reduced to 0.3%. The resulting depth dose fell right between those measured without the shielding. They also agreed with the measurements obtained with a CC13 chamber. Differences with polarity persisted in the buildup region.

Figure 5(b) shows lateral dose profiles measured with and without shielding the internal electrometer. Differences between the opposing polarities decreased when the electrometer was shielded, for example, from 4.4% to 0.2% at a position of 70 mm from the central axis. The resulting curves approached the curve measured with the microDiamond 60019 detector. Due to the offset current, in case of the positive bias and unshielded electrometer, the measured current became negative out of the field. Since Mephysto mc² tbaScan only reports absolute values, the curve appears to increase rather than decrease at approximately 80 mm and further from the field center.

**Figure 6:**
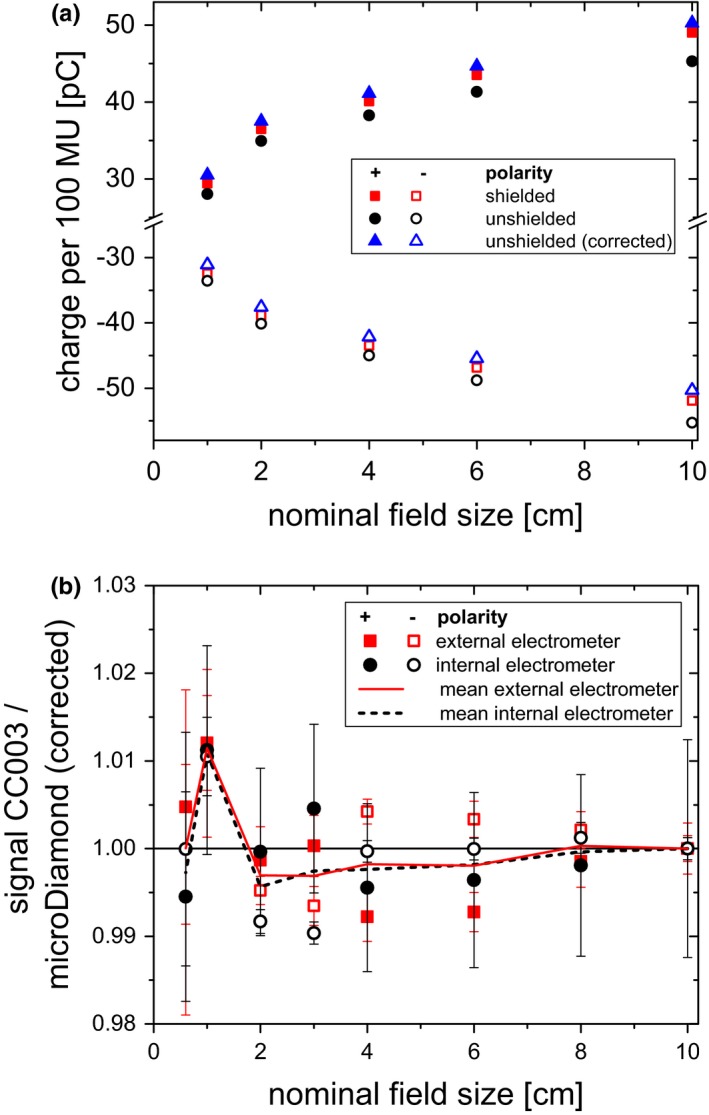
(a) Signals per 100 MU measured with the cc003 chamber centered in fields of different sizes and relative signals normalized to the signal in the 10 × 10 cm² field at 20 cm depth.The internal electrometer was either at EID = 100 cm or at EID = 150 cm and shielded by additional lead. Corrected readings are the unshielded readings corrected by the measured electrometer background for the respective field size. Error bars do not exceed the symbol size and are not shown in (a). (b) Signal ratios of the CC003 chamber normalized to the 10 × 10 cm² field compared to the microDiamond corrected according to TRS 483, at a depth of 10 cm and SSD 90 cm measured with an internal electrometer at EID = 150 cm and an external electrometer. Error bars (one standard deviation) are shown in (b).

The results of output factor measurements are shown in Fig. 6. The measured current for 100 MU increased in all fields and for both polarities when the shielding was added [Fig. 6(a)]. The corrected values, that is, the unshielded measurements corrected by the measured electrometer offset, were slightly above the shielded ones, indicating that the shielding had a large effect but did not entirely protect the electrometer from irradiation. Small systematic deviations between measurements with an internal and an external electrometer were also visible for the normalized signals [Fig. 6(b)]. After averaging between the polarities, there were no noticeable differences between the ratios obtained with an internal and external electrometer. The CC003 chamber signal ratios for the external electrometer were within 1.1% of the microDiamond 60019 detector readings corrected according to TRS 483^4^ down to the smallest field. TRS 483 lists correction factors pooled from several literature sources with an associated uncertainty of the correction factors of approximately 0.01 (k = 2) down to 1 × 1 cm² field size for the microDiamond 60019 detector as stated in its appendix. This uncertainty was not included into the uncertainty budget for the data in Fig. 6. Polarity corrections k_Pol_ for the CC003 chamber averaged over all field sizes were 1.007 (±0.004) for measurements with the external electrometer and 1.029 (±0.003) for measurements with the unshielded internal electrometer.

## 4 DISCUSSION

An electrometer inside the room was shown to produce a current that was reduced with shielding and distance from isocenter, indicating the current is due to stray radiation interacting within the electrometer (Fig. 2). Approximately, the same current was measured with a Farmer type and a thimble type ionization chamber, suggesting that the signal is independent of the detector connected to the electrometer.

With decreasing detector size, depth dose curves measured with those detectors deviated further from curves obtained with plane‐parallel chambers or large ionization chambers, which are assumed to produce the correct curve (Fig. 4). What seems like a polarity problem at first sight can be explained by the currents induced by scattered radiation hitting the electrometer: When this negative background current is added to the signal of the detector, the behavior is dependent on the sign of the measurement signal. For positive polarity, the measurement signal is positive, so the constant current is always subtracted. When one normalizes the measurements, one gets a steeper depth dose curve than without the extra current. For negative polarity, the measurement signal is negative, so the additional background current always increases the absolute signal. Consequently, normalization will yield a shallower depth dose curve. This is exactly what is seen in Fig. 4. It also explains the observation that the apparent polarity effect is reduced when the electrometer is shielded by lead (Figs. 5 and 6).

For output factor measurements, the situation is a little more complex. The dose to the electrometer is not fixed, but also shows a field size dependence. As a consequence, the effect is partly mitigated when normalizing to a reference field. Nevertheless, systematic deviations are introduced into the measurements, for example, in the form of different apparent polarity correction factors k_Pol_. There are differences between the signal ratios with internal and external electrometers [Fig. 6(b)]. Again, the effect vanishes when results obtained with positive and negative polarity are averaged.

While it was not the main purpose of this investigation to obtain correction factors in small fields, it was observed that the CC003 chamber measured with the external electrometer agreed with the corrected microDiamond 60019 detector within 1.1% down to the smallest 0.6 × 0.6 cm² field. The microDiamond 60019 detector is known to over‐respond at very small field sizes. The correction factor according to TRS483 is 0.968 for a 0.6 × 0.6 cm² 6 MV field.^4^ According to the comparison to the corrected microDiamond 60019 detector output [Fig. 6(b)], the CC003 chamber requires output correction below 1%. As a comparison, the suggested Monte Carlo calculated correction obtained for a Gamma Knife Perfexion with 8 mm collimator at 5 cm depth in water is 1.004 for the CC003 chamber.^11^

The observation that the effect increases for smaller chambers can be explained by the ratio of the actual detector signal to the offset current produced by the electrometer. Typical currents induced by the electrometer were of the order of 1 pA for the 100 cm distance. For a large volume chamber with a typical sensitivity of about 21 nC/Gy (FC65), the effect becomes negligible. For the smallest chamber used in this study, the CC003 chamber, the sensitivity is about 0.11 nC/Gy, that is, 190 times smaller.

Depth dose curves with small chambers have been found to disagree with the measurements of larger chambers before. Reggiori et al. described a slight overresponse with increasing depth of the CC003 chamber that increased with increasing field size with a maximum value of 1.9% in a 20 × 20 cm² field.^1^ Unfortunately, polarity was not investigated, a different phantom (iba Bluephantom2) was used and the electrometer was not specified. The observed field‐size dependence agrees with our measurements. Polarity effects for micro‐ionization chambers were also observed by Sarkar et al.^2^, who found up to 5% deviation between depth dose curves measured at different polarities for a 7 mm³ chamber. They checked scanning systems of different manufacturers and external electrometers, concluding that the internal electrometer contributes a signal constant with depth and detector position, although they could not localize the cause of the anomaly. They stated differences between the scanning systems provided by different manufacturers, which seems natural as the phantoms were of different shapes leading to different scatter conditions. In addition, the electrometers of the different scanning systems are at different positions with respect to the phantom, either attached to the phantom or detached with a very long controller cable. Their internal construction details and shielding further contribute to the differences.

Radiation reaching the internal electrometers should be reduced as much as possible. Some manufacturer manuals advise placing the electrometer as far away from the radiation source as the cables allow. The supplied cables might not always be sufficiently long to reach the necessary distance depending on the measurement conditions, for example, with very small chambers or when carrying out off‐axis measurements. To our knowledge, there is no explicit suggestion or consideration of possible problems due to an internal electrometer in dosimetry protocols. Under some conditions, measurements with small ionization chambers can lead to wrong data being introduced into the treatment planning system. Therefore, we would like to stress the suggestions by Sarkar et al. that one should thoroughly check if measurement results seem to depend on the sign of the bias voltage used.^2^ For output factor measurements, external electrometers should be used to avoid the introduction of systematic uncertainties. For profile acquisition, the equipment might not allow the use of an external electrometer. In principle, one could determine the current and correct the measured data accordingly (Fig. 6). One should keep in mind that the current from the internal electrometer depends on the dose it receives, so it cannot be quantified generally and will increase the uncertainties. It needs to be assessed individually for each electrometer type, position and orientation, radiation quality, field size, and scattering object. The effect seems to be reproducible between different electrometers of the same type. In general, if an internal electrometer needs to be used with a detector that produces a low signal, it can be suggested to place the electrometer as far away from the radiation source as possible and to use additional shielding as needed. The exact amount of necessary shielding depends on the measurement task. In this work, the chosen lead bricks were found to be thick enough for all measurements except those out of field, where detector currents are much lower. It may also be sufficient to shield parts of the electrometer. For our electrometer model, the radiation‐sensitive parts are only close to the connectors for the cables leading to the detectors. The remaining effect should be checked for by measuring both polarities and comparing to point measurements taken with an external electrometer or by quantitating the offset with the detector outside the treatment room. The observation might not be limited to small ionization chambers, but could also become apparent with any other low‐sensitivity detector.

While apparent discrepancies between the depth dose curves at positive and negative bias voltage could be explained and reduced at depth with additional shielding, differences near the surface remain (Fig. 5) and are currently being investigated. Polarity effects just below the surface have been analyzed by McEwen et al. for a 30 MV beam.^9^ They found a depth‐dependent effect that is small except close to the surface for the studied Exradin A16 chamber (7 mm³) and suggested electron contamination to be the cause. For electron beams, polarity effects seem to depend on a variety of parameters such as the cable, stem length, energy and field size.^12^ This suggests that the interplay between polarity, detector construction details and electron contamination near the surface needs to be further analyzed.

## 5 CONCLUSIONS

It was shown how an internal electrometer's response to scattered radiation influences relative dose measurements. A current induced by radiation reaching the electrometer results in distorted relative dose ratios becoming apparent when measuring with both polarities. The effect increases when the detector signal decreases and becomes visible when the measurement signal is of an order comparable to the electrometer offset current. In a typical measurement setup in a 10 × 10 cm² field, M20/M10 values measured with a CC003 chamber deviated more than 7% from the value obtained with larger chambers.

To prevent the introduction of erroneous data into the treatment planning system, special care must be taken when small ionization chambers are used in combination with an internal electrometer. We recommend to always test at both polarities and to compare some results to point measurements carried out with an external electrometer or to quantitate the effect of scattered radiation on the electrometer for the specific setup used. To reduce the influence of scattered radiation on the electrometer, shielding the electrometer with lead may be a sufficient, practical solution in many cases.

## CONFLICTS OF INTEREST

There are no conflicts of interest.
